# Psychological assessment of AI-based decision support systems: tool development and expected benefits

**DOI:** 10.3389/frai.2023.1249322

**Published:** 2023-09-25

**Authors:** Katharina Buschmeyer, Sarah Hatfield, Julie Zenner

**Affiliations:** ^1^Faculty of Business, Augsburg Technical University of Applied Science, Augsburg, Germany; ^2^Faculty of Liberal Arts and Science, Augsburg Technical University of Applied Science, Augsburg, Germany

**Keywords:** AI-based decision support systems, work, human-centered evaluation, survey inventory, system properties, characteristics of the supported task, psychological load

## Abstract

This study aimed to develop an evaluation tool that assesses the use of AI-based decision support systems (DSSs) in professional practice from a human-centered perspective. Following the International Organization for Standardization, this perspective aims to ensure that the use of interactive technologies improves users' psychological load experience and behavior, e.g., in the form of reduced stress experience or increased performance. Concomitantly, this perspective attempts to proactively prevent or detect and correct the potential negative effects of these technologies on user load, such as impaired satisfaction and engagement, as early as possible. Based on this perspective, we developed and validated a questionnaire instrument, the Psychological Assessment of AI-based DSSs (PAAI), for the user-centered evaluation of the use of AI-based DSSs in practice. In particular, the instrument considers central design characteristics of AI-based DSSs and the corresponding work situation, which have a significant impact on users' psychological load. The instrument was tested in two independent studies. In Study 1, *N* = 223 individuals were recruited. Based on the results of item and scale analyses and an exploratory factor analysis, the newly developed instrument was refined, and the final version was tested using a confirmatory factor analysis. Findings showed acceptable-to-good fit indices, confirming the factorial validity of the *PAAI*. This was confirmed in a second study, which had *N* = 471 participants. Again, the CFA yielded acceptable-to-good fit indices. The validity was further confirmed using convergent and criterion validity analyses.

## 1. Introduction

Professionals have to make various decisions during the course of their work. For example, asset managers must choose between various investment options, whereas lawyers have to decide on a possible defense strategy for a particular case. For a decision to be made, a conscious and voluntary choice must be made among several alternative courses of action by comparing, considering, and evaluating them based on available data, information, and knowledge (Büssing et al., 2004; Rau et al., 2021). Owing to the growing amount of data and information in our increasingly digitalized and globalized world, decision-making processes have become very complex across various professions (Latos et al., 2017; van Laar et al., 2017; Timiliotis et al., 2022). For many, keeping a track of all relevant new data and information when making decisions and placing them in the context of existing knowledge poses a great challenge (Timiliotis et al., 2022). The amount of available data in some areas has become so vast that it cannot be processed by humans, as it simply exceeds their information processing capacity (Koltay, 2017; Saxena and Lamest, 2018; Shrivastav and Kongar, 2021). Moreover, in everyday work, highly complex decision-making situations are often complicated by stressors, like an elevated time and performance pressure. Such challenges and, for many professionals, overstraining decision-making situations lead to higher levels of uncertainty, stress, and lower decision quality (Phillips-Wren and Adya, 2020). They also affect, for example, job satisfaction (Nisar and Rasheed, 2020) and organizational productivity (Miller and Lee, 2001; Vosloban, 2012).

Although the intensification of digitization and globalization leads to increased risks for companies and professionals, it also opens up new opportunities. For example, the accumulation of data, both in terms of quantity and quality, has enabled impressive improvements in the field of artificial intelligence (AI), which helps in the development of extremely powerful algorithms (Nicodeme, 2020). They are often based on machine-learning models, which are more scalable and flexible than traditional statistical models (Rajula et al., 2020), making them appropriate tools for today's dynamic and complex work environments. For problems such as those described above, researchers have acknowledged the particular great potential of the use of AI-based decision support systems (DSSs; see Brynjolfsson et al., 2011; Cai et al., 2019; Shin, 2020; Tutun et al., 2023), which are often referred to as *Augmented Intelligence Systems* (Jarrahi, 2018; Hassani et al., 2020; Walch, 2020; Kim et al., 2022). As the name suggests, these systems are designed to augment, and not replace, humans in complex decision-making situations by taking over specific task components, like processing big data, which are difficult for human intelligence to handle. In professional practice, this looks like this: AI-based applications analyze the huge amounts of data and information available and make hidden patterns in the data accessible to humans in the form of insights or concrete recommendations for action (Konys and Nowak-Brzezińska, 2023). Humans are free to decide whether to follow the system's recommendation. Thus, humans remain the central element in the interpretation and verification of AI-based systems, resulting in complex decision-making situations and continued sovereignty over the final decisions and associated actions (Hellebrandt et al., 2021). This is pivotal because even though there are powerful algorithms behind AI-based DSSs, they also have limitations and weaknesses like overfitting, lack of transparency, and biases (Pedreschi et al., 2019). Humans can compensate for these weaknesses through their inherent strengths and mental acumen (e.g., critical thinking, creativity, and intuition; Spector and Ma, 2019; Wilkens, 2020). Hence, the introduction of augmented intelligence systems ideally leads to a synergetic interaction between human and machine intelligence, which helps professionals to better handle increased cognitive demands (Kirste, 2019). Consequently, they feel appropriately challenged and less burdened in work-related decision-making situations (Cai et al., 2019), which is reflected, for example, in their higher task performance (Li et al., 2021). From a business perspective, the improved decision-making process should, for example, lead to increased company's performance (Brynjolfsson et al., 2011). To summarize, the introduction of an AI-based DSS should create a mutually beneficial scenario for professionals and their companies.

However, it has been noted that many AI initiatives have failed to achieve their objectives. This can be attributed to several reasons, including technical challenges like insufficient databases, organizational failures like inadequate expectation management, and failed system design. For example, users often cannot find a new system that is sufficiently useful or transparent, making them unwilling to use the system (Westenberger et al., 2022). To avoid this, since 2019, the International Organization for Standardization (ISO) has advocated the adoption of a human-centered design approach in the development of interactive systems such as AI-based DSSs. The approach “aims to make systems usable and useful by focusing on users, their needs and requirements, and applying knowledge and techniques from the fields of human factors/ergonomics and usability. This approach increases effectiveness and efficiency; improves human wellbeing, user satisfaction, accessibility, and sustainability; and counteracts the potential negative effects of use on human health, safety, and performance” (ISO International Organization for Standardization, 2019). To achieve this, the ISO International Organization for Standardization (2019) recommends an organizations' active user involvement throughout the development process and follows a four-step design process: (1) understanding and describing the context of use, (2) specifying user requirements, (3) developing design solutions, and (4) evaluating the design solutions. The fourth step of evaluation plays a decisive role in this process. Here, the success of the project is determined; if not successful, stakeholders can study the concrete modification measures required and process steps that must be repeated. However, when assessing success, the ISO International Organization for Standardization (2019) also underpins the importance of observing not only whether system introduction has led to the intended effects but also whether possible negative, unintended side-effects have occurred.

Based on previous project reports, it is evident that it is common for the introduction of AI-based systems to lead to negative, unintended side-effects. For example, in a case study in the banking sector, Mayer et al. (2020) observed that the introduction of an AI-based system in the lending department led to a perceived loss of competence and reputation among system users. To derive appropriate actions in cases where unintended outcomes occur and in those where desired outcomes are not achieved, it is necessary to gain an accurate understanding of the impact of a new system (and its individual characteristics) on the relevant work situation and its users. This consideration is particularly important when an AI-based DSS is assisting with a core activity, and showcases that the introduction of AI-based DSSs carries particular weight in influencing the user's load experience—both in desirable and undesirable ways. A well-known example of this is related to service and customer support professionals, whose core activity is dealing with customer issues on a daily basis. These professionals now increasingly have access to AI-based DSSs that assist them with a relatively high degree of automation: it provides them with concrete suggestions for actions regarding the requests made by customers. In this scenario, the need for a thorough and comprehensive evaluation of system implementation is undeniable. However, practical evaluation instruments considering this holistic perspective are currently lacking. The available assessment methods comprise either (a) user experience surveys, which enable the evaluation of the impact of specific system properties on users (e.g., SUS, Bangor et al., 2008; Perceived Usefulness and Ease of Use scales, Davis, 1989; meCue, Minge et al., 2017) or (b) job analyses, which allow a comprehensive examination of the influence of new technologies as a whole on task design (e.g., WDQ, Morgeson and Humphrey, 2006; TBS-GA(L), Rudolph et al., 2017; FGBU, Dettmers and Krause, 2020). However, to the best of our knowledge, there is currently no specific practical assessment tool for evaluating the use of AI-based DSSs and that effectively combines both levels of consideration (i.e., user experience and job analyses). Therefore, this study aims to close this gap by developing and validating a questionnaire instrument that not only captures the properties of an AI-based DSS but also the characteristics of the corresponding work situation, thus providing a holistic evaluation framework.

## 2. Conceptualization and use of the evaluation instrument

The newly developed evaluation questionnaire, called Psychological Assessment of AI-based DSSs (*PAAI*), is based on the core idea of many occupational psychology models (e.g., the job demand control model, Karasek, 1979; the Stress-Strain model, Rohmert., 1984; the Transactional Model of Stress and Coping, Lazarus and Folkman, 1984; and the Action Regulation theory, Hacker, 1978). These models describe that when assessing work tasks, a distinction should be made between *work characteristics* (trigger factors) and the resulting *psychological load* (trigger reactions) experienced by professionals. Work characteristics include all identifiable aspects of a task, such as complexity, social environment, and work equipment, which affect human engagement in the task. The immediate impact of psychological work characteristics on an individual, considering one's internal (e.g., intelligence) and external resources (e.g., social support), is referred to as psychological load. Depending on the alignment between psychological work characteristics and individual resources, the psychological load can be positive (e.g., activation and flow experience) or negative (e.g., mental overload and stress). Persistent psychological load has medium- and long-term consequences, including positive outcomes, like satisfaction and wellbeing, or negative outcomes, like dissatisfaction and reduced performance (ISO International Organization for Standardization, 2019).

**Figure 1:**
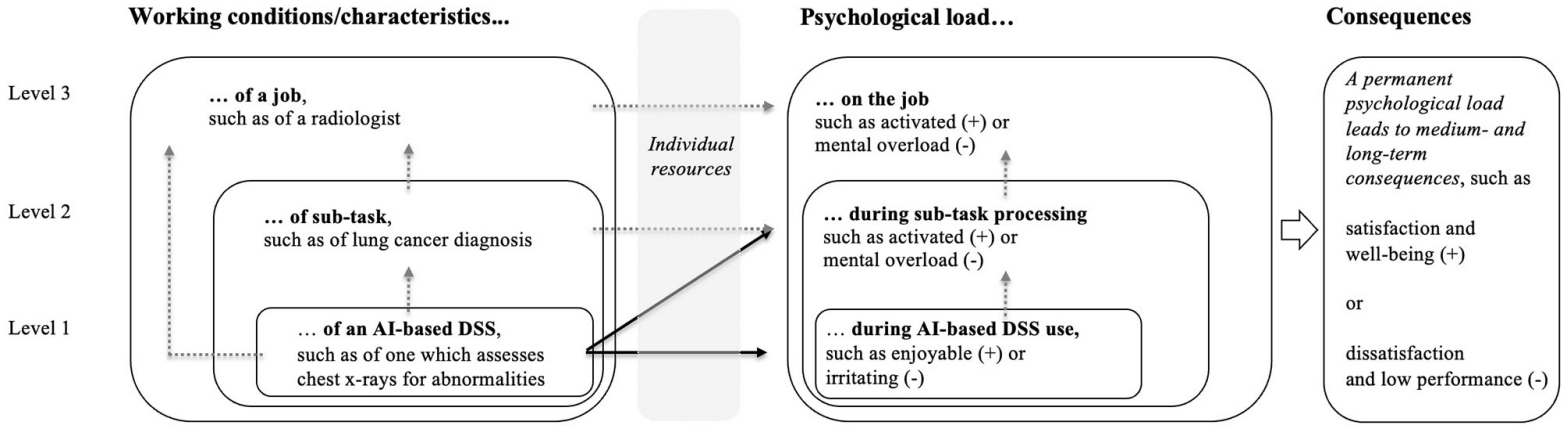
The basic model of the assessment instrument psychological assessment of AI-based decision support systems (PAAI).

From the above-mentioned perspective, the introduction of a new work tool (e.g., an AI-based DSS) can be perceived as a new work task characteristic that should help to alleviate user psychological overload. Following Hacker (1978) hierarchical levels of technology-based tasks, this impact on psychological load can occur at three levels (see Figure 1).

First, the immediate interaction with the system can trigger a psychological response in the user. Depending on the design of the system and the user's resources (e.g., technical knowledge), this can be positive (e.g., enjoyment), or negative (e.g., irritation). Previous research on conventional information systems and DSSs show which design features are particularly influential for user experience (see Venkatesh and Davis, 2000; Calisir and Calisir, 2004; Alshurideh et al., 2020). These findings also apply to AI-based DSSs (see Henkel et al., 2022; Meske and Bunde, 2022). However, since they differ from conventional DSSs in their probabilistic nature and black-box character (Zhang et al., 2020; Jussupow et al., 2021), related design features must also be considered in this case. Section 2.1 provides an overview of the traditional as well as the specific characteristics that current research suggests promote positive human-augmented intelligence interactions.

Second, the implementation of AI-based DSSs can influence users' psychological load regarding the entire processing of the task supported by the system. To this end, they are implemented to support their users in task processing and to handle other—in this case unfavorable—work characteristics (e.g., information overload). Thus, DSSs can and should reduce, for example, user uncertainty and stress in task processing. DSSs based on AI methods are currently seen as particularly promising tools for this purpose. This is because their extremely powerful algorithms enable them to capture both highly complex and dynamic problems (Kirste, 2019; Kim et al., 2022). However, it is likely that the great power of the systems will not only have a direct impact—by providing immediate support in decision-making situations—but also an indirect impact on the psychological load of users. Because they also have a great potential—targeted or untargeted—to change the character of the supported work tasks from the professionals' point of view, which in turn determines their load experience. This kind of change is desirable when, for example, it enables professionals to perceive previously overwhelming tasks as less complex and therefore less stressful. On the other hand, unintended consequences can occur if, for example, employees perceive fewer opportunities for further learning and, as a result, the personality-enhancing aspect of the work activity is lost.

Third, to make the levels of consideration complete, the introduction of an AI-based DSS should also be considered from the overall job perspective. This is because the introduction of an AI-based DSS can also potentially affect cross-task work characteristics, consequently affecting the psychological load of users in relation to their jobs as a whole. For example, recent research shows that professionals can feel threatened in their jobs by the introduction of new technologies (Gimpel et al., 2020); for example, this may occur if new technologies cause them to perceive their jobs as less future-proof (Lingmont and Alexiou, 2020).

To sum up, the introduction of an AI-based DSS can influence the psychological load of users (and the associated consequences) at three different levels: (1) during immediate human-AI interaction, (2) during processing of the supported task, and (3) during executing the entire job. Since the three levels are interrelated, they should all be considered in an evaluation of these systems. During the evaluation, it may turn out that the users' psychological load or related consequences on one or more levels deviate from the desired result, or it may be grasped that further optimization potential remains to be identified. In these cases, it is advisable to first take a closer look at the lowest level, examining its characteristics and derive possible required modification needs. Thereafter, the other levels can be gradually included in the analysis. In this way, the need for action can be identified from a more specific to a more general level.

Moreover, if the various work characteristics are surveyed both as part of a one-time measurement after its introduction and before system introduction, the evaluation can also examine how these have changed as a result of the initiative. Thus, all effects of system introduction—including unintended side-effects—can be precisely tracked and easily corrected if necessary. In practice, this means that the system evaluation can be applied both as part of a one-time measurement or in the form of a pre-post measurement, depending on the exact purpose of the evaluation. In the next section, we provide discussions on level-specific characteristics that (a) have a significant impact on the psychological load of professionals and (b) are closely associated with the implementation of AI-based DSSs. They form the assessment measures in the *PAAI*.

### 2.1. Level 1: human-AI-interaction

At the finest level of consideration, the focus is on the individual design characteristics of AI-based DSSs that strongly influence users' psychological load during their interactions with the system and their willingness to use it in the first place. The best-studied and most important characteristics that all types of information systems should satisfy—and thus the characteristics that are most consistently evaluated—are *Perceived Usefulness* and *Perceived Ease of Use*, as described in the Technology Acceptance Model (Davis, 1989). When evaluating these two factors for AI-based DSSs, it is also important to consider the unique properties of these systems, which significantly influence user perceptions of *Perceived Usefulness* and *Perceived Ease of Use*.

More specifically, the *Perceived Usefulness* of a system is strongly influenced by its information quality (Machdar, 2019) and thus, in the case AI-based DSSs, by the accuracy of the system. This is because, as mentioned earlier, AI-based DSSs operate on a probabilistic basis, meaning that there is no absolute guarantee that a system result will be correct (Zhang et al., 2020). Thus, in order for users to experience systems as valuable and trustworthy, they must provide correct results with the highest possible probability (Shin, 2020). Therefore, when evaluating the *Perceived Usefulness* of AI-based DSSs, it is important to ask directly about the perceived quality of the system output, in addition to considering other conventional and concrete design features, such as the task-technology fit of the system (Goodhue and Thompson, 1995).

The *Perceived Ease of Use* of an AI-based DSSs, as with conventional DSSs, is generated by design features such as self-descriptiveness (ISO International Organization for Standardization, 2020) and simplicity (Lee et al., 2007). AI-based DSSs should also pay particular attention to ensuring that the system output is presented in a way that is easy to understand (Henkel et al., 2022). The aforementioned probabilistic nature of AI systems can make it difficult for users to correctly interpret system output; research in cognitive psychology has shown that humans often have difficulty correctly understanding probabilities, which lead to increased misjudgments (Anderson, 1998). Therefore, to facilitate good user interactions, it is necessary to create intuitively designed interfaces and present results in a human-centered way to reduce the risk of misinterpretation.

In addition to the peculiarity of the probabilistic nature and associated uncertainty of AI-based DSSs, they differ from conventional systems that they develop their own programming rules. Their algorithmic mechanisms for model generation are therefore not transparent (Jussupow et al., 2021). As a result, the underlying logic of these systems is often referred to as a black-box model (Kraus et al., 2021). The lack of information about why an AI-based system operates in a certain way also complicates the interpretation of system outputs. Therefore, an increasing number of AI-based DSSs provide additional explanations using Explainable AI (XAI) methods. These relate to how an AI-based system arrives at its output and what goes into that output (Arrieta et al., 2020). However, a failure to sufficiently *perceive these explanations as comprehensible* can negatively impact trust and acceptance of AI-based DSSs (Shin et al., 2020), as well as the cognitive effort required for decision-making (Meske and Bunde, 2022).

Freely accessible AI assistance systems, like ChatGPT (https://chat.openai.com) and DeepL (https://www.deepl.com/translator), are increasingly bringing AI to the forefront of public awareness. Simultaneously, our own interactions with these cloud-based solutions highlight the criticality of their reliable accessibility, particularly in hectic working situations. Frequent unavailability (e.g., due to overwhelming user demand) can lead to stress (Körner et al., 2019). Therefore*, Perceived Availability* is also a central influencing factor of user experience.

Table 1 provides an overview of what existing research has revealed about the influences of the four system characteristics—*Perceived Usefulness, Perceived Ease of Use, Perceived Comprehensibility*, and *Perceived Availability*—on users' psychological loads. Below, a detailed description of each construct considered in the inventory is presented.

**Table 1:** PAAI's Level 1 assessment criteria and their influence on professionals' psychological load.

**Evaluation criteria by system level**	**Associated results on …**	
	**… professionals' experience of human-machine-interaction (Level 1)**	**… professionals' psychological load during task processing (Level 2)**
Perceived usefulness (PU)	Attitude toward use (Alhashmi et al., 2020); behavioral intention to use a system (Venkatesh and Davis, 2000; Alhashmi et al., 2020; Al Shamsi et al., 2022); technology trust (Amin et al., 2014); technology satisfaction (Amin et al., 2014); actual usage (Rigopoulos et al., 2008)	Decision quality (Wook Seo et al., 2013); performance (Omar et al., 2019; Arnold et al., 2020); engagement (Lackey et al., 2016); workload (Lackey et al., 2016)
Perceived ease of use (PEU)	Attitude toward use (Alhashmi et al., 2020); behavioral intention to use a system (Venkatesh and Davis, 2000; Alhashmi et al., 2020; Al Shamsi et al., 2022); technology trust (Amin et al., 2014); technology satisfaction (Amin et al., 2014); actual usage (Rigopoulos et al., 2008)	Performance (Omar et al., 2019); mental effort (Lackey et al., 2016); frustration (Lackey et al., 2016)
Perceived comprehensibility (PC)	Technology trust (Shin, 2020, 2021; Liu et al., 2022); perceived value (Liu et al., 2022); perceived quality of advice (Gaube et al., 2023)	Performance (Stowers et al., 2020; Gaube et al., 2023); workload (Mercado et al., 2016); cognitive effort (Meske and Bunde, 2022)
Perceived availability (PA)	Stress (Körner et al., 2019); perceived value (Baldauf et al., 2020; Prakash and Das, 2020)	Stress (Körner et al., 2019)

*Perceived Usefulness* refers to the extent to which an individual believes that using an AI-based DSS improves decision-making effectiveness and efficiency (Davis, 1989; Krieger and Lausberg, 2021). To perceive a system as useful, a high task suitability must be perceived by the users, which implies that the system meets the specific requirements of the task it supports (Goodhue and Thompson, 1995; Klopping and McKinney, 2004). Furthermore, the system must provide high-quality information and deliver accurate, timely, complete, and relevant results (Gorla et al., 2010; Hsiao et al., 2013; Atta, 2017; Machdar, 2019).

*Perceived Ease of Use* encompasses the extent of effortlessness of use of an AI-based DSS as perceived by individuals (Davis, 1989). To achieve this, systems should be designed with clear functions and user-friendly interfaces to ensure that the system output is easy to understand (Doshi-Velez and Kim, 2017; Iriani and Andjarwati, 2020; Sati and Ramaditya, 2020). Additionally, simplicity should be prioritized in system design, which can be achieved through the reduction, organization, integration, and prioritization of system features (Lee et al., 2007).

*Perceived Comprehensibility* includes individuals' perceptions of the extent of their clear understanding of reasons for the output generated by the system (Coussement and Benoit, 2021). To achieve this, it is advantageous to provide both general model explanations, which elucidate functional relationships between the input and output variables, and specific explanations, which aid in understanding individual data-related outputs (Kraus et al., 2021). The users should not be overwhelmed with excessive system details; instead concise and effective information should be offered that enables them to effectively utilize the system within their task environment (Mercado et al., 2016).

*Perceived Availability* encompasses the extent to which individuals perceive the content of a system as reliably accessible and retrievable. Usually, this is affected by factors such as the frequency of unexpected system updates, system crashes, error messages, and technical problems (Körner et al., 2019).

### 2.2. Level 2: AI-supported task

At the second evaluation level, along with the system characteristics discussed at Level 1, specific task characteristics of the supported tasks were considered. To facilitate the evaluation process, three broad groups of task characteristics were identified: requirements, resources, and stressors (Iwanowa, 2006). The task characteristics considered in the inventory for each of these groups are discussed in detail below, and they have the following common traits: (a) they have a significant impact on the psychological load of professionals and (b) it is very likely that the introduction of AI-based DSSs will affect them directly or the way professionals interact with them (see Table 2).

**Table 2:** PAAI's Level 2 assessment criteria and their influence on professionals' psychological load, and the potential influence of AI-based DSSs on these criteria.

**Characteristics group**	**Evaluation criteria on task level**	**Associated outcomes on professionals' psychological load and load consequences**	**Possible positive intended effect through system implementation**	**Possible negative unintended effect through system implementation**
Requirements	Perceived Complexity and decision-making requirements (PCDR)	Stress (Phillips-Wren and Adya, 2020); Performance (Maynard and Hakel, 1997; Mosaly et al., 2018; Chinelato et al., 2019); mental effort (Mosaly et al., 2018); satisfaction (Morgeson and Humphrey, 2006)	With AI-based system support, professionals probably perceive tasks/decisions as less complex or feel more confident in dealing with them (Mayer et al., 2020; Wanner, 2021). As a result, they feel less stressed (Cai et al., 2019; Lee et al., 2021). The improved data-driven decision basis is also expected to improve decision quality and performance (Li et al., 2021; Wanner, 2021).	There is the risk of automation bias, in that users may come to often rely on the system's advice and not critically reflect on it (Skitka et al., 1999; Mayer et al., 2020; Panigutti et al., 2022). They may then perceive the task as not complex enough or monotonous, which can lead them to feel under challenged and bored (Loukidou et al., 2009).
	Perceived cooperation and communication requirements (PCCR)	Stress (Zeffane and McLoughlin, 2006); mental health (Lu and Argyle, 1991); happiness (Lu and Argyle, 1991)	If employees feel more confident in decision-making situations with system support, the need for cooperation with colleagues is likely to decrease. If these were previously perceived as too high and high losses of time and energy were associated with them, this can have a positive effect on the experience of psychological load.	If employees feel more confident in decision-making situations with system support, the need for exchange with colleagues is likely to decrease. If these were previously perceived as appropriate, this can be perceived as negative, since social exchange reduced, for example.
Resource	Perceived Latitude for activity (PLA)	Work engagement (Dettmers and Krause, 2020); satisfaction (Morgeson and Humphrey, 2006); motivation (Glaser et al., 2015); loss of irritation (Glaser et al., 2015; Dettmers and Krause, 2020); loss of psychosomatic complaints (Dettmers and Krause, 2020)	No effects are expected.	The introduction of new technologies like AI is often accompanied by process standardization (Silva and Gonçalves, 2022), which in turn probably limit professionals' perceived latitude for activity.
	Perceived use of qualifications and learning opportunities (PUQL)	Satisfaction (Rowden and Conine, 2005); engagement (Jin and McDonald, 2017); intention to stay (Steil et al., 2020)	No effects are expected.	There is a risk that by using AI-based DSSs, professionals rely little on their own skills and thus lose their expertise over time. Since there is no maintenance learning and they do not take advantage of learning opportunities (Mayer et al., 2020).
Stressors	Perceived information overload (PIO)	Irritation (Dettmers and Krause, 2020); stress (Misra et al., 2020); tension (Theron, 2014); tiredness (Theron, 2014); loss of job satisfaction; (Theron, 2014); decision quality (Hwang and Lin, 1999)	Through the use of AI-based DSSs that bundle and process information, the information overload should perceived to be less or its handling easier due to the new resource (Maes, 1995; Aussu, 2023).	Often, stressors influence psychological load to such an extent that they can overshadow other work characteristics (Phillips-Wren and Adya, 2020). Therefore, there is an AI-based DSSs will have little or no impact on professionals' psychological load experience if they remain too high.
	Perceived lack of information (PLI)	Irritation (Dettmers and Krause, 2020); psychosomatic complaints (Dettmers and Krause, 2020)	By using the system, it is likely for fewer information deficits to occur, as the system generates new patterns, new information, and insights from the data (Haefner et al., 2021).	
	Perceived time and performance pressure (PTPP)	Irritation (Dettmers and Krause, 2020); psychosomatic complaints (Dettmers and Krause, 2020); exhaustion (Syrek et al., 2013); loss of work–life balance (Syrek et al., 2013)	It can be assumed that the use of AI-based DSSs alleviates the time and performance pressure experienced by professionals. This is primarily attributed to the system enabling employees to make faster and more confident decisions. Alternatively, a similar level of pressure may be perceived, but the associated challenges can be better managed by professionals through the use of the aforementioned technology (Wanner, 2021; Tutun et al., 2023).	
	Perceived qualification deficits (PQD)	Irritation (Dettmers and Krause, 2020); psychosomatic complaints (Dettmers and Krause, 2020); loss of work engagement (Dettmers and Krause, 2020)	It is likely that AI-based systems compensate for existing skill deficiencies of professionals (Gaube et al., 2023) consequently reducing the experience of negative load consequences.	

#### 2.2.1. Requirements

The group of *requirements* includes all work characteristics that professionals must meet in order to successfully and effectively perform their work tasks. Therefore, requirements are inherent to the nature of the task and unavoidable. In general, the characteristics of this group are considered positive and beneficial for personality development *per se*, but only as long as they fit individual resources of the jobholder. Otherwise, it leads to psychological underload or overload (Iwanowa, 2006; Semmer and Zapf, 2018). Two requirements were considered in the developed inventory: *Perceived Complexity and Decision-making Requirements and Perceived Cooperation and Communication Requirements*.

*Perceived Complexity and Decision-making Requirements* refer to the perceived level of mental demand of a task. It can be categorized into various levels, ranging from routine activities with rehearsed mental requirements to activities requiring productive thinking and problem-solving (Hacker, 2016). Decision-making is a component of complex tasks, and its degree can be assessed by various measures like the number of variables involved (Stemmann and Lang, 2014).

*Perceived Cooperation and Communication Requirements* involve the perceived need to inform and coordinate with colleagues. It includes factors like the duration of communication, number of partners involved, mode of communication (direct or indirect), and content, like information sharing, instruction dissemination, and collaborative problem-solving (Richter et al., 2014). These requirements are often accompanied by highly complex tasks, as they often necessitate cooperation and collaboration between different specialists or departments owing to the diverse skills and knowledge required (Helquist et al., 2011).

#### 2.2.2. Resources

The group of *resources* includes all work characteristics that provide opportunities for action and may or may not be used voluntarily (Zapf, 1998; Semmer and Zapf, 2018). However, the professionals should be aware of these possibilities. Resources have a predominantly positive relationship with the indicators of maintaining and promoting health and fostering personal development (Iwanowa, 2006). In the evaluation tool, the two resources of *Perceived Latitude for Activity and Perceived Use of Qualifications and Learning Opportunities* should be considered and explained.

*Perceived Latitude for Activity* is a multidimensional construct that includes the perceived scope of action, design, and decision-making in a professional set-up. Scope of action refers to the range of available action-related options, including the choice of approach, resources, and temporal organization of task components. This defines the degree of flexibility in performing subtasks in a professional scenario. Design latitude refers to the ability to design processes independently based on goals. Decision-making latitude considers the degree of decision-making authority in task definition and delineation and determines the degree of autonomy associated with an activity (Ulich, 2011).

*Perceived Use of Qualifications and Learning Opportunities* refers to the perception that one can optimally utilize own existing expertise, skills, and abilities professionally. Therefore, a process of learning maintenance occurs. Conversely, low levels of this resource indicated unlearning (Büssing et al., 2004). Learning opportunities are closely related to the use of own qualifications and resources in executing job responsibilities. Interestingly, the existence of learning opportunities can only be assessed by comparing existing and required knowledge, skills, and abilities (Rau et al., 2021).

#### 2.2.3. Stressors

The group of *stressors* encompasses all factors that impede the achievement of task goals and those that require professionals to make additional efforts or take additional risks. These efforts and risks, in turn, increase their work load, time, and effort (Büssing et al., 2004; Semmer and Zapf, 2018). Thus, dealing with stressors has adverse effects on the mental health of most professionals (Iwanowa, 2006). In the *PAAI*, four stressors are considered: *Perceived Information Overload, Perceived Lack of Information, Perceived Time and Performance Pressure, and Perceived Qualification Deficits*.

*Perceived Information Overload* involves the perception of the need to consider or evaluate an amount of information that is larger than the one's information intake and processing capacity (Dettmers and Krause, 2020). Furthermore, it has been noted that humans have a unique characteristic: the more information we are offered, the more information we think we need (Krcmar, 2011). According to Heinisch (2002), this leads to a paradox in knowledge society. This states that in the midst of the flood of information, there is a lack of information.

*Perceived Lack of Information* indicates that information is perceived as missing, unavailable, or not up to date (Dettmers and Krause, 2020).

*Perceived Time and Performance Pressure* describes the perceived imbalance between three work components, as follows: quantity, time, and quality (Trägner, 2006). The mismatch between these three components lies in the fact that a certain amount of work cannot be accomplished in the required or necessary quality in the available working time (Schulz-Dadaczynski, 2017).

*Perceived Qualification Deficits* indicate that, from the perspective of professionals, the work task assigned to them does not match their existing qualifications; these qualifications include technical competencies (e.g., specialized knowledge, work techniques, skills, and abilities) and social and communicative competencies required for the proper execution of a task (Richter et al., 2014). However, the mismatch can be attributed to qualification deficits for an activity, for example, due to insufficient training; as a result, workers feel overtaxed. Low qualification adequacy can also be seen when workers perform activities below their qualification level, triggering a qualitative underchallenge (Dettmers and Krause, 2020).

### 2.3. Level 3: overall job

The third level considers the workplace's cross-task characteristics, which extend beyond task-related aspects and affect users' psychological load in relation to their job. As mentioned earlier, in AI implementation projects, there is a risk that the introduction of AI-based systems may induce a higher level of *Perceived Job Insecurity* among its users. This fear is related to concerns about job loss owing to automation or insufficient proficiency in using digital technologies and media (Gimpel et al., 2020). According to a recent study by Lingmont and Alexiou (2020), professionals who are highly aware of AI and robotics tend to perceive a higher level of job insecurity than those with lower awareness. The implementation of AI-based DSSs probably increases users' awareness of AI, which in turn could raise concerns related to job insecurity and thereby increase psychological load.

## 3. Scale and item generation and qualitative review

To measure the 13 characteristics described (see Section 2), we developed the respective *PAAI*. To be able to observe the impact of the constructs on the professionals in the context of an evaluation, it is also necessary to collect appropriate indicators of the professionals' psychological load and related consequences. Fortunately, several scales already exist for this purpose (e.g., NASA-TLX, Hart and Staveland, 1988; irritation scale, Mohr et al., 2006; stress experience, Richter, 2000), from which a particular variable can be selected as per project objectives. However, one exception is the measurement of psychological load during immediate human-AI interaction. To the best of our knowledge, no questionnaires are available for this variable as of yet. Therefore, we developed an additional scale to measure user *Irritation during System Use*. In developing the 14 scales, we took care to keep the number of statements per scale as short as possible while ensuring that a minimum of three items met the scientific validity criteria (Mvududu and Sink, 2013). The items were formulated using generic terms, so that they can be applied to different occupations and types of AI-based DSSs. Simultaneously, the assessment incorporates design recommendations to facilitate the identification of specific causes and derivation of appropriate action measures.

To test the clarity and face validity of the developed items, cognitive pretests were conducted with *N* = 10 individuals without prior experience of AI-based DSSs in an occupational context. First, a paraphrasing method was used (Porst, 2013). In this method, participants were asked to reproduce the individual statements of the questionnaire in their own words. If a respondent did not understand a statement or understood it incorrectly, the statement was rephrased, clarified with examples, or removed. The revised items were then tested in the second step with the remaining respondents. In this step, a sorting technique was used to examine how respondents assigned the given items to the given constructs. It could be said that the sorting technique is a type of factor analysis that does not require previously collected data (Porst, 2013). All items assigned to the correct category by at least 75% of raters were retained. This development process resulted in a questionnaire with 59 items (Table 1; Supplementary material).

## 4. Empirical testing of the developed items and scales

The newly developed items and scales were empirically tested in two consecutive studies using causal samples. Adjustments like the deletion of items were made as required. Study 1 began with an analysis of the items and scales. Subsequently, an exploratory factor analysis (EFA) was conducted to examine the factor structure of the questionnaire derived from the item and scale analysis. The resulting factor structure, which was expected to be statistically and theoretically adequate, was subjected to confirmatory factor analysis (CFA) for further validation, employing the maximum likelihood (ML) estimation method, and allowing the factors to correlate (detailed information is accessible in the provided data on OSF—see the data availability statement). Study 2 tested the factorial validity of the final model from Study 1 using CFA, utilizing maximum likelihood estimation, with a different sample size. Furthermore, the final scale was assessed in terms of its convergent and predictive validity. In both cases, correlations with other variables collected simultaneously using established instruments were analyzed. Correlation analyses, item and scale analyses, and EFA were conducted using IBM SPSS software version 26.0. For the CFA, RStudio software (version 4.2.0) was used.

### 4.1. Study 1

#### 4.1.1. Method

##### 4.1.1.1. Participants and procedures

On January 24, 2023, *N* = 250 participants from the UK were recruited from a crowdsourcing platform, Prolific, for the survey. The prerequisite was that the participants must being employed and regularly use a DSS in the job. For the survey, it did not matter whether the DSS is based on AI methods because the properties surveyed can be assessed for all DSSs, regardless of which technical solutions are behind them. After excluding participants who, for example, missed one of the two attention checks or had superficial response patterns, *N* = 223 participants remained (*n* = 132 female, *n* = 91 male). Most respondents (42.6%) were aged between 30 and 39 years and worked in customer service and support (12.1%), organization, data processing and administration (11.7%), and marketing and sales (11.2%). Regarding educational level, the most had a bachelor's degree (46.6%).

##### 4.1.1.2. Materials

The survey comprised the newly developed instrument (see Supplementary Table 1) and general demographic questions (e.g., age, gender, and field of activity). Aside from the general demographic questions, all other items were answered on a 5-point scale ranging from 1 (doesn't apply at all) to 5 (applies completely).

#### 4.1.2. Analysis and results

##### 4.1.2.1. Item and scale analysis

In the context of item and scale analysis, it is first checked whether the items are too easy, too difficult, or insufficiently differentiated, and then the internal consistency of the scales is assessed. Specifically, items with a difficulty index between *P* = 0.20–0.80 and a discriminatory power of *rit* ≥ 0.40 are targeted (Bortz and Döring, 2016; Kalkbrenner, 2021). All but two items (19 and 52) met these criteria, and the two items that did not meet the criteria were therefore deleted. Supplementary Table 2 presents the descriptive statistics for each scale at the end of the item and scale analyses.

##### 4.1.2.2. EFA

In the next step, the scales or items were further tested separately within their corresponding levels, as described herein: (1) human-AI interaction, (2) AI-supported task, and (3) overall job (as described in Section 2). We conducted an EFA for each. The prerequisites for the EFA needed to be examined further. First, inter-item correlations were checked; that is, whether each item correlated with at least three other items with a value between *r* = 0.20 to *r* = 0.85. Moreover, we tested whether each item had a Kaiser-Meyer-Olkin (KMO) measure of ≥0.70, Bartlett's test for sphericity of *p* < 0.05, and a measure of sampling adequacy (MSA) >0.8 (Kalkbrenner, 2021; Zhang et al., 2021). These conditions were fulfilled in all cases. To determine the most suitable number of factors for summarizing the elements within each level, two analyses were conducted: principal component analysis (PCA) and parallel analysis (Kalkbrenner, 2021). The results in Supplementary Table 3 indicate that different criteria suggest different numbers of factors.

In subsequent EFAs, the modeling process employed principal axis factorization (PFA) with the oblique rotation method Promax because of the assumed inter-factor correlations (Moosbrugger and Kelava, 2008). The initial starting point for each analysis was the highest assumed number of factors, as shown in Supplementary Table 3, to ensure maximum information preservation. Throughout the exploration, individual items were systematically excluded, and after each exclusion, the PFA was recalculated. Items were excluded if at least one of the following three conditions was not met: First, items should exhibit a factor loading of at least λ ≥ 0.40. Second, it is desirable that the items have no cross-loadings, that is, they should have no loadings of λ ± 0.40 on two or more factors. Meanwhile, if an item does demonstrate cross-loadings, but the loading on one factor is λ ≥ 0.10 higher than on the other factors, researchers can use their theoretical understanding to decide whether the variable should be assigned to a factor or deleted. Third, from a theoretical perspective, all items should fit the respective factors to which they were assigned according to the EFA (Korner and Brown, 1990; Kalkbrenner, 2021). Consequently, of the 28 items included in the EFA at Level 1 (human-AI interaction), seven items (items 1, 5, 9, 12, 13, 17, and 22) were deleted. At Level 2 (AI-supported task), of the 26 items included in the EFA, eight items (items 32, 36, 39, 40, 47, 48, 49, and 50) were deleted. According to the criteria mentioned above, Item 30 should also have been deleted as it loaded highly on two factors (difference λ = 0.05). However, this was not done because of (a) strong theoretical reasons and (b) the decision is statistically confirmed in the subsequent CFA because the model quality is better with the inclusion of the item in the scale than without it.

For the remaining 21 items at Level 1, a five-factor structure resolved a total of *R*^2^ = 60.29% of the variance. In terms of content, the five factors were consistent with the theory described in Section 2. However, at Level 2, contrary to the theoretical assumption, a six-factor structure—not an eight-factor structure—was determined for the remaining 18 items, and it explained *R*^2^ = 60.12% of the cumulative variance. Specifically, the EFA results indicated that the items developed to measure the constructs of decision-making and complexity requirements should be combined with those representing the construct of information overload into a single factor. This merger was supported by theoretical considerations. Moreover, all items related to the construct “lack of information” were deleted because of their high cross-loadings. This deletion was theoretically justifiable and appropriate. For the three remaining items at Level 3, as theoretically hypothesized, we obtained a one-factor structure that resolved *R*^2^ = 68.95% of the variance. A comprehensive depiction of the factor structure encompassing the items and their corresponding factor loadings for each scale is provided in Table 3.

**Table 3:** Overview of exploratory factor analysis and confirmatory factor analysis results in Study 1.

**Level 1: Human-AI-interaction**				**Level 2: AI-supported task**				**Level 3: Overall job**			
**Factor**	**Item**	**Load EFA**	**Load CFA**	**Factor**	**Item**	**Load EFA**	**Load CFA**	**Factor**	**Item**	**Load EFA**	**Load CFA**
PU	2	0.74	0.75	PCDR	30	0.70	0.80	PJI	57	0.89	0.89
	3	0.82	0.79		31	0.72	0.71		58	0.89	0.89
	4	0.77	0.78		45	0.69	0.62		59	0.70	0.70
	6	0.78	0.77		46	0.71	0.63			
	7	0.75	0.77	PCCR	33	0.80	0.83				
PEU	8	0.76	0.76		34	0.77	0.76				
	10	0.77	0.74		35	0.88	0.84				
	11	0.70	0.67	PLA	37	0.57	0.59				
	14	0.74	0.77		38	0.78	0.67				
	15	0.74	0.75		41	0.72	0.77				
PC	16	0.83	0.76	PUQL	42	0.73	0.75				
	18	0.71	0.70		43	0.73	0.76				
	20	0.69	0.70		44	0.86	0.81				
	21	0.69	0.74	PTPP	51	0.75	0.75				
PA	23	0.59	0.62		53	0.76	0.76				
	24	0.78	0.76	PQD	54	0.87	0.87				
	25	0.78	0.78		55	0.83	0.83				
	26	0.77	0.76		56	0.77	0.77				
ISU	27	−0.89	0.89								
	28	−0.91	0.91								
	29	−0.82	0.82								
**Model fit indices:**				**Model fit indices:**				**Model fit indices:**			
χ^2^/*df* = 1.48				χ^2^/*df* = 1.48				χ^2^/*df* = 0			
CFI = 0.97				CFI = 0.97				CFI = 1.00			
TLI = 0.96				TLI = 0.96				TLI = 1.00			
RMSEA = 0.05				RMSEA = 0.05				RMSEA = 0.00			
SRMR = 0.04				SRMR = 0.05				SRMR = 0.00			

N = 223.

χ^2^, Chi squared; df, degree of freedom; CFI, Comparative Fit Index; ISU, Irritation during System use; PA, Perceived Availability; PC, Perceived Comprehensibility; PCCR, Perceived Cooperation and Communication; PCDR, Perceived Complexity and Decision-making Requirements; PEU, Perceived Ease of Use; PJI, Perceived Job Insecurity; PLA, Perceived Latitude for activity; PQD, Perceived Qualification deficit; PTPP, Perceived Time and performance pressure; PU, Perceived Usefulness; PUQL, Perceived Use of Qualifications and Learning Opportunities; RMSEA, Root Mean Square Error of Approximation; SRMR, Standardized Root Mean Residual; TLI, Trucker–Lewis index.

##### 4.1.2.3. CFA

CFAs were conducted to confirm the factor structure of the developed instrument according to the EFAs. This analysis was used to determine the fit between the model and obtained data (Bandalos, 2002). Following Hu and Bentler (1999), the model fit index was determined using chi-square/degree of freedom (good fit = 0 ≤ χ^2^/*df* ≤ 0.2; acceptable fit = 2 < χ^2^/*df* ≤ 0.3), comparative fit index (CFI; good fit = 0.97 ≤ CFI ≤ 1.00; acceptable fit = 0.95 ≤ CFI < 0.97), Tucker-Lewis index (TLI; good fit = 0.97 ≤ TLI ≤ 1.00; acceptable fit = 0.95 ≤ TLI < 0.97), root mean square error of approximation (RMSEA; good fit = 0 ≤ RMSEA ≤ 0.05; acceptable fit = 0.05 < RMSEA ≤ 0.08), and standardized RMR (SRMR; good fit = 0 ≤ SRMR ≤ 0.05; acceptable fit = 0.05 < SRMR ≤ 0.10). The indices for the three models are listed in Table 3. Acceptable to good fit indices were observed for all models.

### 4.2. Study 2

#### 4.2.1. Method

##### 4.2.1.1. Participants and procedure

From 6 to 16 February 2023, a second survey was conducted in the UK and US via Prolific. The participation criteria were the same as those of Study 1. Of the *N* = 535 participants, *N* = 471 individuals (*n* = 235 female, *n* = 233 male, *n* = 3 non-binary) remained after data cleaning as in the first study. Most respondents (36.5%) were between 30 and 39 years and had a bachelor's degree (39.7%). In addition, most worked in customer service and support (12.1%), organization, data processing and administration (8.1%), and marketing and sales (7.9%).

##### 4.2.1.2. Materials

Along with the final questionnaire inventory from Study 1 (Supplementary Table 4) and demographic data, we collected data on load indicators and consequences to test criterion validity. Specifically, system use satisfaction was surveyed through a custom-developed item, “I like using the system” and trust in a system was surveyed using a custom-developed question, “How much do you trust the system?” The subjective stress experienced during a task was surveyed using the six items developed by Richter (2000), which were translated from German to English. Moreover, mental effort and mental exhaustion were assessed with two questions from the BMS short scales, which were translated from German into English (Debitz et al., 2016). Task enjoyment was surveyed with the item “How much pleasure do you usually get from the work task?” Furthermore, competence experience during task processing was assessed using four items adapted from a prior study (Sailer, 2016). Job satisfaction was surveyed using an item, which was translated from German into English, from a past study (Kauffeld and Schermuly, 2011). For later testing of convergent validity, the six-item meCue (Minge et al., 2017), which measures the usefulness and usability of technologies, was used. All statements were answered on a 5-point scale ranging from 1 (doesn't apply at all) to 5 (applies completely). The questions, for example on mental effort and exhaustion, were responded on a slider scale ranging from 1 (low) to 10 (high).

#### 4.2.2. Analysis and results

##### 4.2.2.1. Item and scale analysis

Table 4 shows the descriptive statistics for all the scales. All scales showed an internal consistency of α ≥ 0.70 (Hussy et al., 2013), and the *Perceived Time and Performance Pressure* scale a Spearman-Brown coefficient of 0.757. In addition, high mean values and low standard deviations were observed for most scales.

**Table 4:** Results of the item and scale analysis in Study 2.

**Level**	**Characteristics or load**	**Scale**	**Number of items**	**Range of the scale**	**Cronbach's α**	**Mean value**	**Standard deviation**
1	System characteristics	*PU*	5	1;5	0.84	3.85	0.64
		*PEU*	5	1;5	0.84	3.67	0.69
		*PC*	4	1;5	0.77	3.38	0.73
		*PA*	4	1;5	0.83	3.63	0.77
		meCue	6	1;5	0.86	3.72	0.67
	Load indicators	*ISU*	3	1;5	0.88	2.22	0.85
2	Task characteristics	Satisfaction with system use	1	1;5	-	3.50	0.92
		Trust in the system	1	1;10	-	7.15	1.60
		*PCDR*	4	1;5	0.72	3.20	0.78
		*PCCR*	3	1;5	0.87	2.96	1.01
		*PLA*	3	1;5	0.76	2.94	0.90
		*PUQL*	3	1;5	0.82	3.21	0.90
		*PTPP*	2	1;5	0.76	2.95	0.99
		*PQD*	3	1;5	0.76	2.19	0.87
	Load indicators	Stress experience	6	1;5	0.86	2.09	0.74
		Mental effort	1	1;10	-	6.76	1.89
		Mental exhaustion	1	1;10	-	5.35	2.17
		Task enjoyment	1	1;10	-	5.54	2.25
		Competence experience in task processing	4	1;5	0.77	3.66	0.69
3	Job characteristics	*PJI*	3	1;5	0.88	2.23	1.02
	Load indicators	Job satisfaction	1	1;10	-	6.92	2.08

N = 471.

ISU, Irritation during System use; PA, Perceived Availability; PC, Perceived Comprehensibility; PCCR, Perceived Cooperation and Communication; PCDR, Perceived Complexity and Decision-making Requirements; PEU, Perceived Ease of Use; PJI, Perceived Job Insecurity; PLA, Perceived Latitude for activity; PQD, Perceived Qualification deficit; PTPP, Perceived Time and performance pressure; PU, Perceived Usefulness; PUQL, Perceived Use of Qualifications and Learning Opportunities.

PAAI criteria are shown in italics.

##### 4.2.2.2. CFA

To test the factor structure of the final version of the *PAAI* on another sample, a second CFA was conducted for each Level and for the overall instrument. As in Study 1, the results showed acceptable to good fit indices (Hu and Bentler, 1999) for all three models per Level, and for the overall model (see Table 5). A detailed overview of the individual factor loadings is provided in Supplementary Table 4.

**Table 5:** Confirmatory factor analysis results from Study 2.

	**χ^2^/*df***	**CFI**	**TLI**	**RMSEA**	**SRMR**
Good fit	0 ≤ χ^2^/*df* ≤ 0.2	0.97 ≤ CFI ≤ 1.00	0.97 ≤ TLI ≤ 1.00	0 ≤ RMSEA ≤ 0.05	0 ≤ SRMR ≤ 0.05
Acceptable fit	0.2 < χ^2^/*df* ≤ 0.3	0.95 ≤ CFI < 0.97	0.95 ≤ TLI < 0.97	0.05 < RMSEA ≤ 0.08	0.05 < SRMR ≤ 0.10
**Models tested**					
Level 1 scales	1.76	0.97	0.97	0.04	0.04
Level 2 scales	2.04	0.96	0.95	0.05	0.04
Level 3 scales	0	1.00	1.00	0.00	0.00
All scales	1.45	0.96	0.96	0.03	0.04

N = 471.

χ^2^, Chi squared; df, degree of freedom; CFI, Comparative Fit Index; RMSEA, Root Mean Square Error of Approximation; SRMR, Standardized Root Mean Residual; TLI, Trucker–Lewis index.

##### 4.2.2.3. Correlation analysis

A correlation analysis was conducted to further test the validity of the *PAAI* (see Supplementary Tables 5, 6). In this analysis, the Level 1 scales were correlated with the meCue (Minge et al., 2017) to test their convergent validity and overall summary. It showed that all Level 1 scales as a whole correlate strongly with the meCue (*r* = 0.83, *p* < 0.01) according to Cohen's (1988) criteria (*r* ≥ 0.10 small, *r* ≥ 0.30 moderate, *r* ≥ 0.50 strong effect). From the perspective of individual scales, the *Perceived Usefulness* (*r* = 0.70, *p* < 0.01) and *Perceived Ease of Use* (*r* = 0.83, *p* < 0.01) showed particularly very high correlations, as expected, and the *Perceived Comprehensibility* (*r* = 0.54, *p* < 0.01) and *Perceived Availability* (*r* = 0.56, *p* < 0.01) showed high correlations.

Moreover, for all levels, the collected criterion-related variables capturing the load indicators and the corresponding predictive scales were found to be correlated. These results demonstrate the criterion-related validity of the instrument. Furthermore, in the task characteristic group of requirements, the *Perceived Complexity and Decision-making Requirements* scale was moderately positively related to mental effort (*r* = 0.41, *p* < 0.01) and mental exhaustion (*r* = 0.28, *p* < 0.01), and simultaneously positively related to task enjoyment (*r* = 0.15, *p* < 0.01). Contrastingly, stressors such as *Perceived Qualification Deficits* correlated with negative load indicators like stress experience (*r* = 0.64, *p* < 0.01), and had no significant relationship with positive load indicators, such as task enjoyment (*r* = 0.06, not significant) or competence experience during task processing (*r* = −0.30, *p* < 0.01). This undesirable influence of stressors was also evident at Level 3, with the *Perceived Job Insecurity* scale correlating negatively with job satisfaction (*r* = −0.34, *p* < 0.01). However, variables in the resources group such as *Perceived Latitude for Activity* (Level 2) were moderately correlated with task enjoyment (*r* = 0.40, *p* < 0.01) and competence experience during task processing (*r* = 0.31, *p* < 0.01).

## 5. Discussion

This study aimed to develop and validate an evaluation tool that assesses the use of AI-based DSSs in the workplace, strongly emphasizing the human aspect. Using this human-centered perspective, this study ultimately aimed to ensure that the implementation of new technology has a positive impact on user psychological wellbeing, as well as helps in avoiding unintended negative consequences that could hinder personal development in the workplace. To be able to verify the outcomes of AI-based DSSs implementation, it was necessary to understand the effects of system deployment on users and the associated work situation in a differentiated manner as part of the evaluation. Only in this way can the need for adaptation be specifically derived if necessary.

Thus, an instrument called *PAAI* was developed to capture the following design characteristics in the context of AI-based DSSs: (1) system characteristics of AI-based DSSs that are particularly important from the users' perspective; (2) work-related characteristics of the AI-supported task that are particularly influential for professionals' psychological load and known to play a frequent role in the context of implementation of new technology based on the augmented intelligence approach; and (3) cross-task work characteristics that are often relevant from the professionals' perspective in this context. The selection of the specific system-, task-, and job-related design characteristics collected in the *PAAI* is guided by this research. In total, 13 characteristics were initially identified from the literature and measured with 56 items after an initial, concise, cognitive preliminary study. The newly developed questionnaire was then extensively tested in a preliminary quantitative study, which yielded the necessary adjustments to the instrument. The refined version of the questionnaire was tested in a second quantitative study using another sample. The final instrument encompasses a total of 11 design characteristics measured by 39 items (see Supplementary Table 4).

### Looking at the results

In both studies, the items or scales generated to capture system-, task-, or job-related characteristics were first analyzed in detail within their associated characteristic group or level of consideration. This procedure guaranteed that the newly developed scales function well within their respective levels, and before they are evaluated as a coherent whole across all three levels at the end of Study 2. It also ensured that the three questionnaire parts can be used independently if needed.

In the first section of the questionnaire, in which items assessed the perceived system characteristics of AI-based DSSs from the user's perspective, the EFA in Study 1 yielded a four-factor structure: *Perceived Usefulness, Perceived Ease of Use, Perceived Comprehensibility, and Perceived Availability*. It fit well with the expected theoretical structure (see Section 2). Strictly speaking, however, the EFA resulted in a five-factor structure, as items measuring the psychological load indicator of professionals (i.e., in the form of *Irritation during System Use*) were also included in the analysis. This is because no questionnaire had, thus far, been developed to capture users' direct experiences of psychological load during direct human-AI interactions. Therefore, the inclusion of the *Irritation during System Use* scale in the EFA was necessary to ensure the validity of this newly developed instrument. Furthermore, an exploratory analysis showed that for the items on system characteristics, the EFA results do not differ depending on whether the *Irritation during System Use* scale is included, which indicates the high separability of this dependent variable from the other four independent variables. Contrarily, this construct of independence within the scales of system properties was not as pronounced as shown by the cross-loadings observed in the EFA for the associated items. This finding is not unexpected, as previous studies have shown a close association between individual system characteristics, such as perceived usefulness and ease of use (Suki and Suki, 2011). To delineate the individual constructs more clearly, we eliminated items that could not be clearly assigned to a particular construct. This item reduction procedure was not problematic, as the analysis commenced with an item surplus in order to identify the most powerful and relevant items for the primary study. Therefore, despite the item reduction, all Level-1-scales (a total of five) show good internal consistency, as indicated by the Cronbach's alpha values (Hussy et al., 2013) ranging from α = 0.81–0.91 in Study 1. These acceptable reliabilities are also shown in Study 2, as the Cronbach's alpha values ranged from α = 0.77–0.88.

Importantly, both studies had high mean values (tending to be at the high end of the scale) and low standard deviations for all four system characteristic scales. These observations can be interpreted as an indication of the strong predictive role of the captured system characteristics for system use. This is because participants in both studies evaluated only systems that are regularly used in everyday life. Furthermore, the criterion validity of the developed system characteristic scales on professionals' psychological load experience during immediate human-AI interactions was also empirically confirmed in this study. In particular, in Study 2, all four scales showed, following Cohen's (1988) methodology, moderately negative correlations with the criterion *Irritation during System Use*; thus, both the criterion and construct validity of the newly developed scales were demonstrated. As in Study 1, consistently acceptable goodness-of-fit indices were observed for the final scales in the CFA on a second independent sample. Along with factorial validity, convergent validity was also examined to confirm construct validity. As expected, the *Perceived Usefulness* and *Perceived Ease of Use* scales showed a strong correlation with the *meCue* (Minge et al., 2017), as this well-established instrument maps two very similar constructs. Conforming to this, the *Perceived Comprehensibility* and *Perceived Availability* scales correlated moderately with the *meCue* scale, as with the *Perceived Usefulness* and *Perceived Ease of Use* scales.

The second part was designed to obtain the relevant work-related characteristics of AI-supported tasks in the context of AI-based DSSs. Initially, this questionnaire section comprised eight task characteristics based on the theoretical foundations (see Supplementary Table 1), and had a total of 27 items. However, the assumed factor structure for the newly developed items could not be confirmed in Study 1. The EFA results revealed high cross-loadings between items in the hypothesized scales of *Perceived Complexity and Decision-Making Requirements, Perceived Information Overload, Perceived Lack of Information, and Perceived Time and Performance Pressure*, suggesting the need for adjustment. These findings can be attributed to the fact that work-related characteristics often co-occur in practice and partially influence each other (Dettmers and Krause, 2020; Phillips-Wren and Adya, 2020; Rau et al., 2021).

To ensure the valid measurement of the constructs, two approaches were employed. First, inaccurate items were gradually eliminated during the exploratory phase. This was not problematic, because the preliminary study started with a larger number of items than required. Moreover, whenever reasonable, items from closely related constructs were combined into a single factor. Consequently, during the item deletion process, all items related to the construct of *Perceived Lack of Information* were removed, along with single items from other assumed scales. By removing the entire *Perceived Lack of Information* construct, the relationships among the remaining constructs became clearer. Given that AI-based DSSs are primarily designed to improve the handling of information overload and complex decision processes (Dietzmann and Duan, 2022; Stenzl et al., 2022), and addressing information lack through the identification of novel patterns only yields the possibility of an incidental benefit, we do not consider the omission of this task characteristic of the inventory to be critical. The second approach for improvement involved merging two closely related constructs: *Perceived Complexity and Decision-Making Requirements* and *Perceived Information Overload*. This decision to include the *Perceived Information Overload* scale in the *Perceived Complexity and Decision-making Requirements* is also supported by theoretical considerations since high decision-making and complex requirements often involve dealing with a substantial number of variables and their associated information (Phillips-Wren and Adya, 2020; Rau et al., 2021). From a professional perspective, these two components are likely to be perceived as unified entities rather than as separate constructs. After these adjustments, the CFA results of both the preliminary and main studies consistently showed acceptable to good fit indices for the new six-factor structure of the questionnaire. Furthermore, satisfactory reliabilities were observed for all scales in Studies 1 and 2, ranging from α = 0.72 to α = 0.87. It is noteworthy that although the *Perceived Time and Performance Pressure* scale comprises only two items, it met all the reliability and validity criteria. Since the survey was to be as concise as possible for practical reasons, there was no need to add a third item to the scale, as often recommended by prior studies like that conducted by Mvududu and Sink (2013). In addition to factorial validity, the criterion validity of all the scales was confirmed in the main study. For example, the mental effort criterion showed the strongest correlation with the scale *Perceived Complexity and Decision-making Requirements*. This result is consistent with previous research, showing that complexity and decision demands are significant predictors of cognitive effort (Lyell et al., 2018). Furthermore, the correlation results support those of previous studies, demonstrating a significant relationship between users' negative load experiences and qualification deficits (Dettmers and Krause, 2020). As expected, the results also showed the positive correlation of the two resources variables of *Perceived Latitude for Activity* and *Perceived Use of Qualifications and Learning Opportunities* with indicators of positive load, like task enjoyment.

To holistically evaluate the introduction of an AI-based DSS, the last section of the *PAAI* focuses on a cross-task job characteristic, more specifically, on *Perceived Job Insecurity*. This focus serves to enable the tool to provide data on whether this variable increases from the perspective of the affected professionals as a result of the introduction of new technology. In both the preliminary and main studies, the results consistently confirmed the reliability and construct validity of the scale. Furthermore, the main study proves criterion validity, as this construct correlates negatively with positive load indicators, in this case job satisfaction, in line with previous reports.

Thus, the results of Studies 1 and 2 provide compelling evidence of the validity and reliability of all three parts of the final questionnaire. Furthermore, the construct validity of the questionnaire instrument was assessed in the main study using CFA, showing satisfactory-to-very good fit indices and the overall validity of the instrument.

### 5.1. Limitations and future research

Thus far, the empirical results have confirmed the reliability and validity of the new questionnaire instrument, which can be used both in practice in the context of evaluating AI-based DSSs and in scientific research. This instrument is economic, easy-to-use, and has a solid scientific basis. However, this study has some limitations. First, the validation of the questionnaire relied solely on questionnaire-based instruments; therefore, the results may have been influenced by social desirability bias. Therefore, future validation studies should include a wider range of data sources. For example, the additional assessment of relevant system properties using mathematical metrics, such as system accuracy, is an important sub-design criterion for perceived usefulness (Yin and Qiu, 2021) psychological load indicators using physiological and biochemical measures (Lean and Shan, 2012). Second, because we aimed at developing a questionnaire with the shortest possible survey duration, it is critical to note that convergent validity was only tested for the characteristic scales of the lowest human-AI-interaction level. Scholars are thus recommended to test both the convergent and discriminant validity of the scales at the other two levels (AI-supported tasks and overall jobs) in future studies. This would allow for a more comprehensive assessment of instrument validity across all system levels. Third, data collection for this study was conducted via a paid crowdsourcing platform, which may raise concerns about data quality (Douglas et al., 2023).

To address these issues, (a) a conscious decision was made to use a sample provider that, according to prior research (Peer et al., 2022), delivers the highest data quality; (b) two control questions were included in the questionnaire used in each of the studies, and the data of all participants who failed one of these questions were immediately excluded. Nonetheless, it is advisable to validate the instrument using a separate sample that does not receive financial compensation for participation as well as that is not exclusively from the US and the UK—but instead from various countries and cultures. This will further strengthen the confidence in the observed results and their generalizability beyond the paid crowdsourcing platform sample. Furthermore, the target group of the questionnaire comprised people who used DSSs in their daily work. We decided to not impose any further specific participation conditions related to the AI methods behind the system for several reasons. First, the *PAAI* can be used separately from this specific technical solution, even if it is simultaneously assumed that, especially in the case of AI-based DSSs, attention must be paid to ensure that systems are designed to be, e.g., sufficiently comprehensible because of their black-box nature. Second, the definition of the subject of AI varies widely, and until this date, there is no universally expected definition of the subject (Alter, 2023). Finally, users may not be aware of the specific technical solutions underlying their DSS. Therefore, by omitting the technical solution, the *PAAI* could focus on capturing user perceptions and experiences of the DSS, rather than their awareness of the underlying AI methods. Nevertheless, future studies should investigate whether the term “AI” alone influences user experience and behavior. Previous research investigating the impact of AI-based DSSs on user psychological load has so far mainly focused on the particular characteristics of accuracy and transparency of these systems (see Stowers et al., 2020; Jacobs et al., 2021; Jussupow et al., 2021; Gaube et al., 2023). These arise, as described in the theory section, from the essence of AI-based DSSs; namely, their probabilistic nature and black-box character. In addition, individual studies have been exploring the effect of the timing of the introduction of AI-generated advices to users (Jussupow et al., 2021; Langer et al., 2021). For example, initial findings suggest that users are more satisfied and experience higher self-efficacy in task processing if they receive support from the system after first independently processing the information underlying their decision-making (Langer et al., 2021). However, further evidence is required before generalizations can be made on this topic. Future research efforts should therefore explore other design and implementation characteristics of AI-based DSSs, including the timing of support, and investigate the influence of the term “AI”. This work can yield deeper understandings of the dynamics of user experience and psychological load in relation to AI-based DSSs. Ultimately, this research may enable the identification of other potential key characteristics that should be considered when evaluating AI-based DSSs. Furthermore, future research could conduct research to identify under which design aspects and contextual conditions users of augmented intelligence systems (e.g., AI-based DSSs) perceive these systems as optimal complements to their own abilities, and how the degree of augmentation affects users' psychological load; for example, regarding their own experience of motivation and empowerment. Finally, it would also be interesting to investigate what inversely influences professionals' experience of load on their perception of the system and work-related characteristics. Previous studies have indicated that professionals' current load experience also influences their perceptions of working conditions (Rusli et al., 2008). To increase the acceptance of users toward a new work system, it could be helpful to implement it not in particularly stressful peak periods but in times of moderate workloads.

### 5.2. Practical implications

In augmented-intelligence projects, it is critical for organizations to prioritize future users throughout the development and validation processes. As mentioned in the introduction, the success of augmented intelligence implementation, like AI-based DSSs, in the workplace ultimately depends on the experience and behavior of the employees involved. If they are not ready to use the new technology, the project is likely to fail during the implementation phase. To avoid this, organizations could follow the four phases of the human-centered design approach when implementing an AI-based DSS (ISO International Organization for Standardization, 2019). This requires the involvement of a transdisciplinary team that includes psychological experts and usual technical experts. The expertise of the former is valuable in tasks like requirements analysis, adaptation of work habits, changes in communication during implementation, and evaluation. In the evaluation, it is not sufficient to assess only whether the intended effects were achieved; it is equally important to identify any unintended effects that may have occurred. To gain insight into why newly implemented systems actually have an impact, organizations could conduct comprehensive surveys to understand their effects on individuals and their work environments. This comprehensive understanding facilitates the development of tailored action plans. Along with the use of the *PAAI* evaluation tool, organizations could consider incorporating complementary criteria of system performance, like accuracy (Kohl, 2012) and response time (Tsakonas and Papatheodorou, 2008). Furthermore, Organizations could also include users' individual resources in the evaluation, considering the stress and strain models (ISO International Organization for Standardization, 2017). For example, their expertise level or AI knowledge (Gaube et al., 2023) could be included to identify users' qualification needs. Moreover, data on project management related variables could be collected. As per prior studies, changes in communication are extremely influential in AI project success, particularly expectation management (Alshurideh et al., 2020). Therefore, organizations could also evaluate the success of specific communication measures and whether any further action is needed.

## 6. Conclusion

This study emphasizes the importance of a human-centered design approach in the development and implementation of augmented intelligence projects, as well as the implementation of a user-centered evaluation within this framework. An evaluation tool suitable for this purpose, called *PAAI*, was developed. The novel instrument can be seen as a holistic tool that, along with immediate interface design, focuses on personality-promoting workplace design. The *PAAI* can not only be used selectively to evaluate the impact of AI-based DSSs implementation projects on users, but also as a starting point for the requirements analysis of the four-step human-centered design process. Thus, it could be used as a pre-post measurement. Thus, organizations can use the *PAAI* to develop AI-supported workplaces that are conducive to a positive mental health among workers. Although the *PAAI* was validated by the two independent studies reported in this manuscript, further research is required to collect data from more diverse samples and verify evidence consistency. Moreover, the use of additional data sources, such as objective and qualitative measures, should help further validate the newly developed instrument.

## Data availability statement

The datasets presented in this study can be found in online repositories. The names of the repository/repositories and accession number(s) can be found below: https://osf.io/vfykx/?view_only=03628f3f25c249668e0770f0fb6f9c6f.

## Ethics statement

Ethical approval was not required for the studies involving humans because the study was a voluntary survey on Decision Support Systems and Working Conditions that did not give rise to an ethics vote (e.g., there was no deception). The studies were conducted in accordance with the local legislation and institutional requirements. Written informed consent for participation was not required from the participants or the participants' legal guardians/next of kin in accordance with the national legislation and institutional requirements because after the information text about the survey, there was a button to agree to the survey. Thus, the consent was only in the form of a click, but without a signature. This was because the survey was completely anonymous and without any form of intervention. Anyone could voluntarily participate in the study and stop at any time.

## Author contributions

KB was responsible for the conception and design of the study, data collection, analysis, interpretation, and was the primary writer of the manuscript. SH accompanied with the conception of the study. SH and JZ assisted with the study design and critically revised the manuscript. All authors approved the final version of the manuscript for submission.
