# Supplementary material for: Psychological assessment of AI-based decision support systems: tool development and expected benefits
Source: Front Artif Intell. 2023 Sep 25;6:1249322. doi: 10.3389/frai.2023.1249322 (PMC10561554; doi:10.3389/frai.2023.1249322)
Supplement: Supplementary file 1 [file Table_1.DOCX]

Supplementary Material

Psychological Assessment of AI-based Decision Support Systems: Tool Development and Expected Benefits

Katharina Buschmeyer*, Sarah Hatfield, Julie Zenner

*** Correspondence:** Katharina Buschmeyer: katharina.buschmeyer@hs-augsburg.de

# Supplementary Tables

## Table 1. First version of the developed questionnaire instrument PAAI before study 1 and 2.

| **Item No.** | **Item** | **Assumed scale** |
| --- | --- | --- |
|  | The system is very useful. | Perceived Usefulness (PU) |
|  | The system creates real added value. |  |
|  | Decisions can be made more quickly, easily, safely or effectively with the aid of the system. |  |
|  | The system output is often an excellent basis for decision-making. |  |
|  | The system output provides valuable information for decision-making. |  |
|  | Decisions can be made efficiently with the aid of the system. |  |
|  | The system is a suitable tool for the work task it supports. |  |
|  |  |  |
|  | The system is easy to operate. | Perceived Ease of Use (PEU) |
|  | The system looks tidy and clear. |  |
|  | The system is designed with simplicity in mind. |  |
|  | The system is self-explanatory at most points. |  |
|  | Most of the system functions and the way they operate are easy to understand. |  |
|  | The system output is easy to understand. |  |
|  | The system output is easy to interpret. |  |
|  | The system output is presented in an easy-to-understand manner. |  |
|  |  |  |
|  | Sufficient information is available on how the data processing in the system works. | Perceived Comprehensibility (PC) |
|  | There is enough information about the general functioning behind the system. |  |
|  | If needed, there is enough information about how the system works in the backend. |  |
|  | Explanations are partly missing to be able to understand the system output in detail. (-) |  |
|  | If needed, the system provides helpful additional information on the system output. |  |
|  | The system provides sufficient additional information and explanations on the system output. |  |
|  | The system displays all necessary additional information about the system output. |  |
|  |  |  |
|  | The use of the system is rarely disrupted by system updates, system crashes or other technical problems. | Perceived Availability (PA) |
|  | The system is always available and ready for use. |  |
|  | One can rely on the system's services to be available at all times. |  |
|  | The system guarantees constant access to its services. |  |
|  |  |  |
|  | I’m often irritated by the system. | Irritation during System Use (ISU) |
|  | I sometimes feel annoyed because of the system. |  |
|  | The system quickly makes me angry. |  |
|  |  |  |
|  | The task often requires me to make very complex decisions. | Perceived Complexity and Decision-making Requirements (PCDR) |
|  | The task requires me to compare, weigh and evaluate a wide variety of information in order to reach a decision. |  |
|  | While doing the task, I often have to give it a great deal of thought. |  |
|  |  |  |
|  | While fulfilling the task, I have to exchange and coordinate a lot with colleagues and managers. | Perceived Cooperation and Communication Requirements (PCCR) |
|  | To successfully complete the task, I have to work together with other colleagues. |  |
|  | The job entails a lot of contact with colleagues. |  |
|  |  |  |
|  | I am relatively free to choose the way I carry out the task. | Perceived Latitude for Activity (PLA) |
|  | The task can be carried out flexibly - e.g. I can use different work tools or determine the work sequence myself. |  |
|  | I can bring my own concepts and ideas to the task. |  |
|  | The task gives me the opportunity to be creative and to implement my own ideas. |  |
|  | In performing the task, I’m able to decide a lot of things for myself. |  |
|  | In performing the task, I have a lot of decision-making freedom. |  |
|  |  |  |
|  | The task offers an excellent opportunity to apply my knowledge and (professional) skills. | Perceived Use of Qualifications and Learning opportunities (PUQL) |
|  | The task offers many learning opportunities. |  |
|  | The task gives me the opportunity to further develop my skills and competences. |  |
|  |  |  |
|  | The task often involves handling very extensive or unstructured information. | Perceived Information Overload (PIO) |
|  | The task involves handling a large amount of information. |  |
|  | In the task, the daily amount of information is very high. |  |
|  |  |  |
|  | In the task, it regularly happens that existing documents and information are incomplete. | Perceived Lack of Information (PLI) |
|  | The task often involves information that is ambiguous or incorrect. |  |
|  | The task frequently requires additional research due to the lack of complete information. |  |
|  |  |  |
|  | There’s a great deal of time pressure when performing the task. | Perceived Time and Performance Pressure (PTPP) |
|  | The amount of work involved in the task is so great that it’s often difficult to complete it all. |  |
|  | The task brings a great deal of pressure to perform. |  |
|  |  |  |
|  | When working on the task, I regularly have the feeling that I am not sufficiently qualified for it. | Perceived Qualification Deficits (PQD) |
|  | I lack some qualifications, such as technical knowledge and working techniques that are necessary for successfully completing the task. |  |
|  | While performing the task, I often have the impression that my qualifications do not match the work requirements. |  |
|  |  |  |
|  | I feel insecure in my job because of ever more advanced technologies, such as artificial intelligence. | Perceived Job Insecurity (PJI) |
|  | I’m worried that, sooner or later, I will lose my job because of new technologies. |  |
|  | Every now and then I ask myself whether I’ll be able to meet the future demands of the technological world of work. |  |

## Table 2. Results of the final item and scale analysis from study 1.

| **Level** | **Scale** | **Number of items** | **Cronbach’s α** | **Mean Value** | **Standard**  **Deviation** | **Skewness** | **Kurtosis** |
| --- | --- | --- | --- | --- | --- | --- | --- |
| **1** | PU | 7 | .91 | 3.92 | .66 | -1.72 | -1.34 |
|  | PEU | 8 | .90 | 3.74 | .68 | -1.32 | -1.10 |
|  | PC | 6 | .87 | 3.47 | .74 | -1.34 | 0.84 |
|  | PA | 4 | .81 | 3.74 | .76 | -4.56 | 2.36 |
|  | ISU | 3 | .91 | 2.20 | .88 | 4.27 | 1.08 |
| **2** | PCDR | 3 | .78 | 3.11 | .88 | -0.11 | -1.56 |
|  | PCCR | 3 | .85 | 3.00 | .96 | 0.44 | -1.27 |
|  | PLA | 6 | .86 | 2.97 | .79 | -0.16 | -1.89 |
|  | PUQL | 3 | .81 | 3.31 | .86 | 1.59 | -0.95 |
|  | PIO | 3 | .73 | 3.31 | .84 | -0.82 | -0.11 |
|  | PLI | 3 | .74 | 2.69 | .82 | 2.14 | -1.05 |
|  | PTTP | 2 | .73 | 2.86 | .93 | 1.47 | -1.14 |
|  | PQD | 3 | .86 | 2.21 | .95 | 3.37 | -1.14 |
| **3** | PJI | 3 | .86 | 2.17 | .97 | 3.31 | -1.64 |

*Note.* *N*=223. Range of the scale 1;5. ISU: Irritation during System use. PA: Perceived Availability. PC: Perceived Comprehensibility. PCCR: Perceived Cooperation and Communication. PCDR: Perceived Complexity and Decision-making Requirements. PEU: Perceived Ease of Use. PJI: Perceived Job Insecurity. PLA: Perceived Latitude for activity. PQD: Perceived Qualification deficit. PTPP: Perceived Time and performance pressure. PU: Perceived Usefulness. PUQL: Perceived Use of Qualifications and Learning Opportunities.

**Table 3.** Proposed factor numbers from principal component analysis and parallel analysis in Study 1.

|  | **KMO** | **Bartlett’s test** | **Factors from** | | | |
| --- | --- | --- | --- | --- | --- | --- |
|  |  |  | **PCA** | | **Parallel Analysis** | |
|  |  |  | **λ > 1** | Scree Plot | **λ > 1** | Scree Plot |
| **Level 1:**  **Human-AI-Interaction** | .939 | (𝑋² (378)=3886.90, *p*<.001 | 5 | 5 | 4 | 5 |
| **Level 2:**  **AI-supported task** | .861 | (𝑋² (325)=2963.10, *p*<.001 | 5 | 7 | 3 | 6 |
| **Level 3:**  **Overall job** | .708 | (𝑋² (3)=338.92, *p*<.001 | 1 | 1 | 1 | 1 |

Note*.* PCA: principal component analysis.

**Table 4**. Finale version of the developed PAAI and related CFA factor loadings.

| **Level** | **Scales** | **Item No.** | **Item** | **Factor Loading** |
| --- | --- | --- | --- | --- |
| 1 | PU | 2. | The system creates real added value. | .70 |
|  |  | 3. | Decisions can be made more quickly, easily, safely or effectively with the aid of the system. | .70 |
|  |  | 4. | The system output is often an excellent basis for decision-making. | .72 |
|  |  | 6. | Decisions can be made efficiently with the aid of the system. | .76 |
|  |  | 7. | The system is a suitable tool for the work task it supports. | .70 |
|  |  |  |  |  |
|  | PEU | 8. | The system is easy to operate. | .70 |
|  |  | 10. | The system is designed with simplicity in mind. | .70 |
|  |  | 11. | The system is self-explanatory at most points. | .63 |
|  |  | 14. | The system output is easy to interpret. | .77 |
|  |  | 15. | The system output is presented in an easy-to-understand manner. | .79 |
|  |  |  |  |  |
|  | PC | 16. | Sufficient information is available on how the data processing in the system works. | .67 |
|  |  | 18. | If needed, there is enough information about how the system works in the backend. | .64 |
|  |  | 20. | If needed, the system provides helpful additional information on the system output. | .66 |
|  |  | 21. | The system provides sufficient additional information and explanations on the system output. | .73 |
|  |  |  |  |  |
|  | PA | 23. | The use of the system is rarely disrupted by system updates, system crashes or other technical problems. | .68 |
|  |  | 24. | The system is always available and ready for use. | .75 |
|  |  | 25. | One can rely on the system's services to be available at all times. | .82 |
|  |  | 26. | The system guarantees constant access to its services. | .75 |
|  |  |  |  |  |
|  | ISU | 27. | I’m often irritated by the system. | .90 |
|  |  | 28. | I sometimes feel annoyed because of the system. | .86 |
|  |  | 29. | The system quickly makes me angry. | .80 |
|  |  |  |  |  |
| 2 | PCDR | 30. | The task often requires me to make very complex decisions. | .69 |
|  |  | 31. | The task requires me to compare, weigh and evaluate a wide variety of information in order to reach a decision. | .63 |
|  |  | 45. | The task often involves handling very extensive or unstructured information. | .58 |
|  |  | 46. | The task involves handling a large amount of information. | .63 |
|  |  |  |  |  |
|  | PCCR | 33. | While fulfilling the task, I have to exchange and coordinate a lot with colleagues and managers. | .80 |
|  |  | 34. | To successfully complete the task, I have to work together with other colleagues. | .86 |
|  |  | 35. | The job entails a lot of contact with colleagues. | .83 |
|  |  |  |  |  |
|  | PLA | 37. | The task can be carried out flexibly - e.g. I can use different work tools or determine the work sequence myself. | .62 |
|  |  | 38. | I can bring my own concepts and ideas to the task. | .84 |
|  |  | 41. | In performing the task, I have a lot of decision-making freedom. | .70 |
|  |  |  |  |  |
|  | PUQL | 42. | The task offers an excellent opportunity to apply my knowledge and (professional) skills. | .75 |
|  |  | 43. | The task offers many learning opportunities. | .78 |
|  |  | 44. | The task gives me the opportunity to further develop my skills and competences. | .81 |
|  |  |  |  |  |
|  | PTPP | 51. | There’s a great deal of time pressure when performing the task. | .74 |
|  |  | 53. | The task brings a great deal of pressure to perform. | .83 |
|  |  |  |  |  |
|  | PQD | 54. | When working on the task, I regularly have the feeling that I am not sufficiently qualified for it. | .78 |
|  |  | 55. | I lack some qualifications, such as technical knowledge and working techniques that are necessary for successfully completing the task. | .68 |
|  |  | 56. | While performing the task, I often have the impression that my qualifications do not match the work requirements. | .69 |
|  |  |  |  |  |
| 3 | PJI | 57. | I feel insecure in my job because of ever more advanced technologies, such as artificial intelligence. | .87 |
|  |  | 58. | I’m worried that, sooner or later, I will lose my job because of new technologies. | .90 |
|  |  | 59. | Every now and then I ask myself whether I’ll be able to meet the future demands of the technological world of work. | .76 |

*Note.* *N*=471. PU= Perceived Usefulness. PEU= Perceived Ease of Use. PC=Perceived Comprehensibility. PA= Perceived Availability. ISU= Irritation during System use PCDR= Perceived Complexity and Decision-making Requirements. PCCR= Perceived Cooperation and Communication. PLA=Perceived Latitude for activity. PUQL= Perceived Use of qualifications and learning opportunities. PTPP=Perceived Time and performance pressure. PQD=Perceived Qualification deficits. PJI=Perceived job insecurity. PAAI criteria are written in italics.

**Table 5.** Correlation matrix for Level 1 latent scales from study 2.

|  | **Variable** | **1** | **2** | **3** | **4** | **5** | **6** | **7** | **8** |
| --- | --- | --- | --- | --- | --- | --- | --- | --- | --- |
| Characteristics | 1. PU |  |  |  |  |  |  |  |  |
|  | 2. PEA | .63** |  |  |  |  |  |  |  |
|  | 3. PC | .53** | .51** |  |  |  |  |  |  |
|  | 4. PA | .53** | .53** | .42** |  |  |  |  |  |
|  | 5. PU+PEA+PC+PA | .84** | .84** | .75** | .77** |  |  |  |  |
|  | 6. meCue | .70** | .83** | .54** | .56** | .83** |  |  |  |
| Load indicators | 7. Satisfaction with system use | .55** | .60** | .38** | .39** | .60** | .63** |  |  |
|  | 8. Trust in the system | .55** | .38** | .36** | .45** | .54** | .52** | .46** |  |
|  | 9. ISU | -.42** | -.50** | -.35** | -.39** | -.52** | -.52** | -.49** | -.42** |

*Note.* *N*=471. ISU: Irritation during System use. PA: Perceived Availability. PC: Perceived Comprehensibility. PEU: Perceived Ease of Use. PU: Perceived Usefulness. * indicates p < .05. ** indicates p < .01.

**Table 6.** Correlation matrix for Level 2 latent scales from study 2.

|  | **Variable** | **1** | **2** | **3** | **4** | **5** | **6** | **7** | **8** | **9** | **10** |
| --- | --- | --- | --- | --- | --- | --- | --- | --- | --- | --- | --- |
| Characteristics | 1. PCDR |  |  |  |  |  |  |  |  |  |  |
|  | 2. PCCR | .47** |  |  |  |  |  |  |  |  |  |
|  | 3. PLA | .24** | .18** |  |  |  |  |  |  |  |  |
|  | 4. PUQL | .37** | .33** | .58** |  |  |  |  |  |  |  |
|  | 5. PTPP | .57** | .38** | .04 | .16** |  |  |  |  |  |  |
|  | 6. PQD | .19** | .23** | .23** | .20** | .25** |  |  |  |  |  |
| Load indicators | 7. Mental Effort | .41** | .26** | .13** | .36** | .34** | .10* |  |  |  |  |
|  | 8. Mental Exhaustion | .28** | .24** | -.06 | .04 | .30** | .23** | .33** |  |  |  |
|  | 9. Stress | .19** | .22** | .02 | .02 | .29** | .64** | .09 | .36** |  |  |
|  | 10. Task Enjoyment | .15** | .12** | .40** | .43** | .06 | .06 | .31** | -.10* | -.14** |  |
|  | 11. Competence Experience | .08 | .04 | .31** | .36** | -.05 | -.30** | .09* | -.24** | -.43** | .38** |

*Note.* *N*=471. PCCR: Perceived Cooperation and Communication. PCDR: Perceived Complexity and Decision-making Requirements. PLA: Perceived Latitude for activity. PQD: Perceived Qualification deficit. PTPP: Perceived Time and performance pressure. PUQL: Perceived Use of Qualifications and Learning Opportunities.* indicates p < .05. ** indicates p < .01.
